# Knowledge, Beliefs, and Practices Towards Cochlear Implantations Among Otorhinolaryngologists in India

**DOI:** 10.1007/s12070-023-03527-5

**Published:** 2023-02-10

**Authors:** Rohit Ravi, Dhanshree R. Gunjawate, Ajay M. Bhandarkar, Krishna Yerraguntla

**Affiliations:** 1 Department of Audiology and SLP, Kasturba Medical College, Mangalore, Manipal Academy of Higher Education, Manipal, Karnataka 575001 India; 2 Department of Otorhinolaryngology, Kasturba Medical College, Manipal, Manipal Academy of Higher Education, Manipal, Karnataka India; 3 King Khalid University, Abha, Saudi Arabia

**Keywords:** Otorhinolaryngologists, Survey, Cochlear implant, India

## Abstract

The outcome of the cochlear implant is dependent highly on the knowledge, belief and practice of cochlear implant in otolaryngologists who are among the important team members. The study explored the knowledge, beliefs, and practices towards cochlear implantations among otorhinolaryngologists in India. An online cross-sectional survey study was carried out using convenient sampling among otorhinolaryngologists in India. Phase-I involved developing and validating of a questionnaire to study the knowledge, beliefs, and practices towards cochlear implants among otorhinolaryngologists in India while phase II involved administration of the questionnaire and analysis. Data collection was conducted using Google Forms. A total of 106 otorhinolaryngologists participated across 24–65 years of age and with experience ranging from 1 to 42 years. The participating otorhinolaryngologists reported having good knowledge about the candidacy for a cochlear implant but having limited knowledge of the recent developments and governmental schemes. The otorhinolaryngologists displayed positive beliefs regarding cochlear implantation. Most recommended a battery of tests to determine the candidacy and gave a lot of importance to rehabilitation (96.2%) and surgery for implantation (83%). The respondents also practiced giving importance to a team approach involving multiple team members. High costs and financial burden emerged to be the major challenges for cochlear implantation in India*.* The findings of the survey indicate an overall positive belief and practices towards cochlear implantation by otorhinolaryngologists in India. However, there is a need to spread more awareness among them about the recent advances and schemes that would further improve their service delivery.

## Introduction

Cochlear implant (CI) is a surgical implantable device that bypasses the damaged cochlea, and provides direct stimulation to auditory nerves, further, the auditory nerves carry the signal to the brain where the signal is recognized. CI is used to restore hearing in children or adults who are severely hard of hearing or deaf who exhibit limited benefit for conventional amplification. Initially, only post-lingual adults with profound deafness were considered suitable for cochlear implant. Later, Food and Drug Administration (FDA) extended its approval of CI for prelingual deaf children up to age 12 months, and many children younger than 12 months are also getting benefits of CI [1]. Recently, Cochlear Limited, received the U.S. Food and Drug Administration approval for lowering the age to 9 months [2].

The CI can benefit in terms of speech perception, acquisition of auditory skills, spoken word recognition and speech intelligibility in case of post lingual adults with profound hearing loss. Whereas, in children with prelingual and those with congenital hearing loss CI can be beneficial for all aspects of communication. Early CI can also help in improved language outcomes, communication abilities, better quality of life, leading to better inclusion into the hearing world [3–5].

Cochlear implant (CI) success depends on multidisciplinary team which consist of otorhinolaryngologists, audiologists, speech-language therapist, psychologists, aural rehabilitation specialists, educational specialists, social workers, neuropsychologist, vocational rehabilitation specialist and family members [1]. There are various barriers reported in literature which can hamper the CI process. Ravi et al. [6] reported various parent reported barriers to CI which included child related, financial constraints, device/surgery related, time constraints, social issues, and service delivery. The professional related barrier includes lack of knowledge, training and familiarity, limited discussion of CIs with patient & further the referral process [7–10].

The otorhinolaryngologists act as the key members of the team and are involved in the diagnosis of sensorineural hearing loss, determining the candidacy for CI, surgical aspect of CI and medical consideration. The knowledge, beliefs, and practices towards cochlear implantations is the most essential factor which will determine the positive attitude towards cochlear implantation and therefore, its recommendation. The present study aimed to explore the knowledge, beliefs, and practices towards cochlear implantations among otorhinolaryngologists in India.

## Method

The study was conducted in accordance with the Helsinki declaration [11]. An online cross-sectional survey study was carried out using convenient sampling. The study as conducted in two phases. Phase I involved developing and validating of a questionnaire to study the knowledge, beliefs, and practices towards cochlear implants among otorhinolaryngologists in India. The phase II involved administration of the questionnaire and analysis.

### Phase I—Questionnaire Development and Validation

A self-reported questionnaire was developed in English language based on expert opinion and existing literature. The developed questionnaire was provided to three experienced experts in the field of otology and audiology. The expert were asked to rate their opinion for each question using a rating scale; not relevant, somewhat relevant, quite relevant, and highly relevant. The questions rated to be quite relevant and highly relevant were included. It was further validated by an otorhinolaryngologist surgeon with over 10 years of experience with cochlear implantation to check for the suitability of the questionnaire. In this manner, content and face validity was carried out. All the recommended changes were included in the final questionnaire. The final questionnaire comprised of subdomains such as demographic and work details, items related to knowledge towards cochlear implantation, surgery, schemes, factors for candidacy, beliefs towards cochlear implantation and practices recommended. The final open-ended question probed the challenges in cochlear implantation in India.

### Phase II—Questionnaire Administration and Data Collection

The questionnaire developed at the end of phase I, was made available as a Google Forms and made accessible via a uniform resource link (URL). The questionnaire started with a brief description about the study followed by a consent form. The participation was voluntary. Only those respondents who consented to participate were directed to the form. Otorhinolaryngologists practicing in India were chosen for the study. The URL was mailed to the otorhinolaryngologists registered with the Association of otolaryngologist’s in India & Cochlear implant group of India. At the end of the survey respondents were thanked for their participation and encouraged to forward the link to other fellow otorhinolaryngologists. All the responses were saved in Google drive and accessible to only the investigators.

### Statistical Analysis

Suitable summary statistics were used for summarizing variables, continuous variables with mean, standard deviation and range and discrete variables with frequency and percentage. Results were graphically represented wherever suitable. Statistical Package for Social Sciences SPSS Version 20 was used for all statistical analysis.

## Results

The study comprised of 106 otorhinolaryngologists, with a mean age 37.56 (± 10.24), range 24–65 years and mean experience of 9.66 (± 9.85) range 1–42 years. The gender distribution was 62 males (58.5%) and 44 females (41.5%). Out of the 106 otorhinolaryngologists, 27 (25.5%) performed CI, ranging from 2 to 500 surgeries till the time of survey. The distribution of qualification of these otorhinolaryngologists were as follows; MS (83.09%), and Resident (16%).

The otorhinolaryngologists were asked about which program by the Government of India supports cochlear implantation. The responses included Assistance to Disabled Persons for Purchase/Fitting of Aids and Appliances (ADIP) scheme (55.7%), National Programme for Prevention and Control of Deafness (37.7%), Rashtriya Bal Swasthya Karyakram (5.7%) and Sound hearing initiative (0.9%). Further, scattered responses were obtained for the lowest age for paediatric cochlear implant for children with bilateral, profound sensorineural hearing loss according to U.S. Food and Drug Administration. These included 3 months (10.4%), 6 months (42.5%), 9 months (20.8%), 12 months (26.4%).

**Fig. 1:**
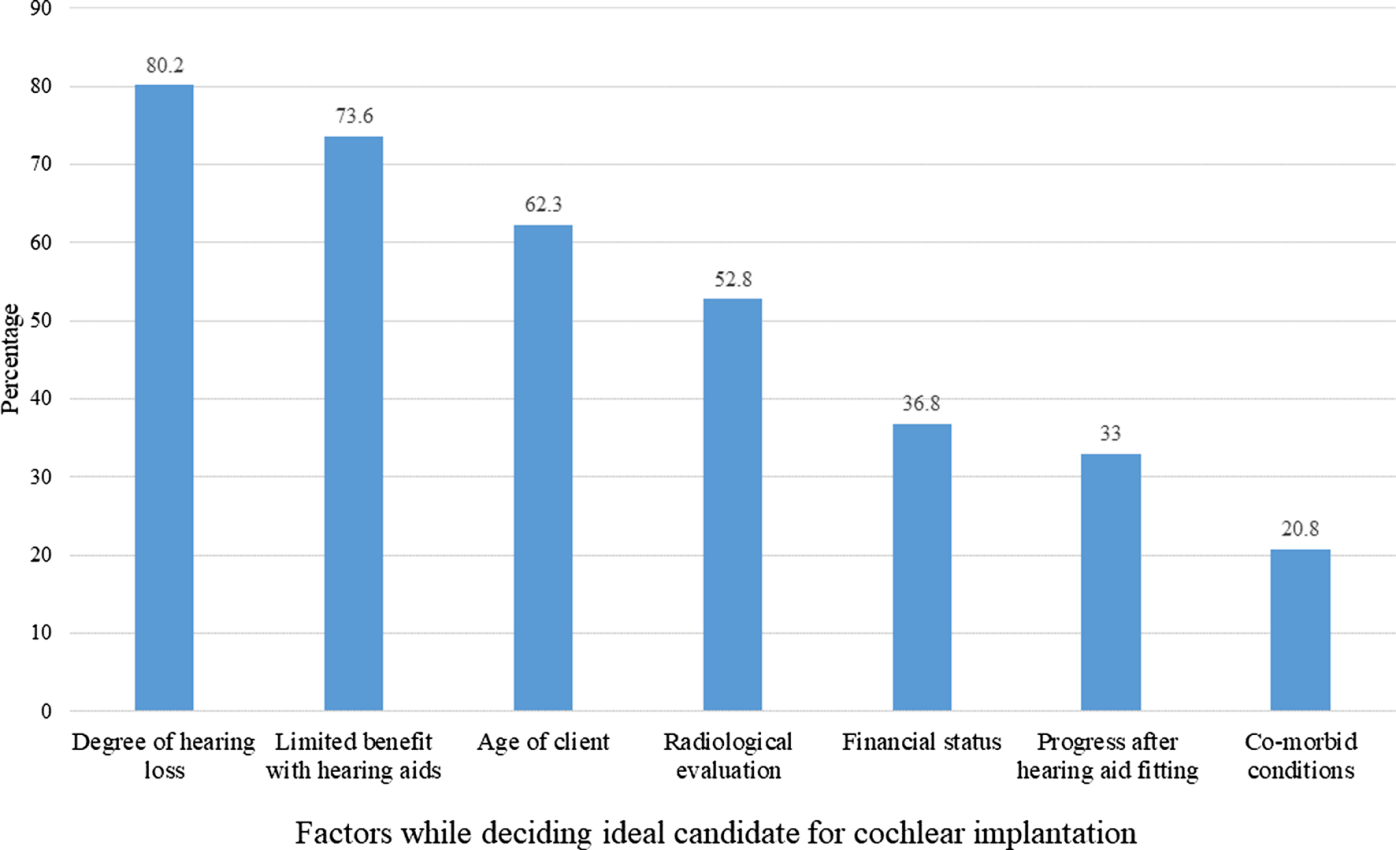
Factors while deciding ideal candidate for cochlear implantation

The beliefs of the otorhinolaryngologists cochlear implantation have been tabulated in Table 1. In a child with congenital bilateral profound sensorineural hearing loss, unilateral implant does not give equally good benefit as a bilateral cochlear implant was felt by 67.9% otorhinolaryngologists. With respect to how often children should get all recommended doses of pneumococcal vaccines before cochlear implant surgery, 63.2% answered 2 weeks before surgery, 16% answered 1 week before surgery while 20.8% were not sure. Further, 63.2% recommended use of one single intravenous antibiotic dose while 36.8% recommended antibiotic cover during entire length of hospital stay. The otorhinolaryngologists were asked to indicate the factors considered while deciding an ideal candidate for cochlear implantation, their responses have been depicted in Fig. 1.

**Table 1:** Beliefs towards cochlear implantation

	Strongly disagree (%)	Disagree (%)	Neutral (%)	Agree (%)	Strongly agree (%)
Cochlear implants require regular maintenance and adjustment	10 (9.4)	19 (17.9)	13 (12.3)	46 (43.4)	18 (17)
Cochlear implant can be a hindrance during sports activities (they slip, get lost, etc.)	9 (8.5)	41 (38.7)	27 (25.5)	27 (25.5)	2 (1.9)
Qualitatively (in terms of hearing sensitivity) there is no difference between hearing aids and cochlear implants	35 (33)	57 (53.8)	8 (7.5)	5 (4.7)	1 (0.9)
Additional training is required in dealing with cochlear implant patients	6 (5.7)	3 (2.8)	10 (9.4)	51 (48.1)	36 (34)

The basic preoperative assessments recommended for a recipient undergoing cochlear implantation included a range of tests and a use of test battery approach. On an average, 7 tests (mean 7.12 ± 2.61) were chosen in the test battery. The most common test chosen was pure tone audiometry (87.7%) followed by auditory brainstem response (81.1%) and computerized tomography scan (80.2%). Additional tests such as speech-language assessments (69.8%) and psychological evaluation (67%). The other tests included magnetic resonance imaging (66%), otoacoustic emission (64.2%) and tympanometry (50%). The less common choices included aided and unaided response (44.3%), cochlear microphonics (37.7%), acoustic reflexes (35.8%), and middle and late latency responses (27.4%).

The otorhinolaryngologists were asked to indicate the three most important steps in cochlear implantation. The most common option included rehabilitation (96.2%), surgery (83%) and evoked compound action potential (ECAP) responses (34.9%). The other steps included hearing preservation (25.5%), electrodes (20.8%), cochlear implant coverage (21.7%), coding strategy (14.2%), ability of an individual to enhance the user’s appreciation to music (2.8%) and remote fitting (0.9%).

61.3% otorhinolaryngologists felt that an otorhinolaryngologist plays an important during switch on, 19.8% did not feel so while 18.9% were not sure. The otorhinolaryngologists were asked according to them; ‘How often should the child follow-up with the operating surgeon during rehabilitation?’ 47.2% asked for a follow-up after every mapping session, 28.3% scheduled a follow-up every month while 24.5% asked for a follow-up whenever there is a medical issue. Little more than half (51.9%) of the otorhinolaryngologists waited for 21 days before the implant is activated, 38.7% waited till the wound heals, 18.9% waited for 10 days while 3.8% waited for 5 days.

**Fig. 2:**
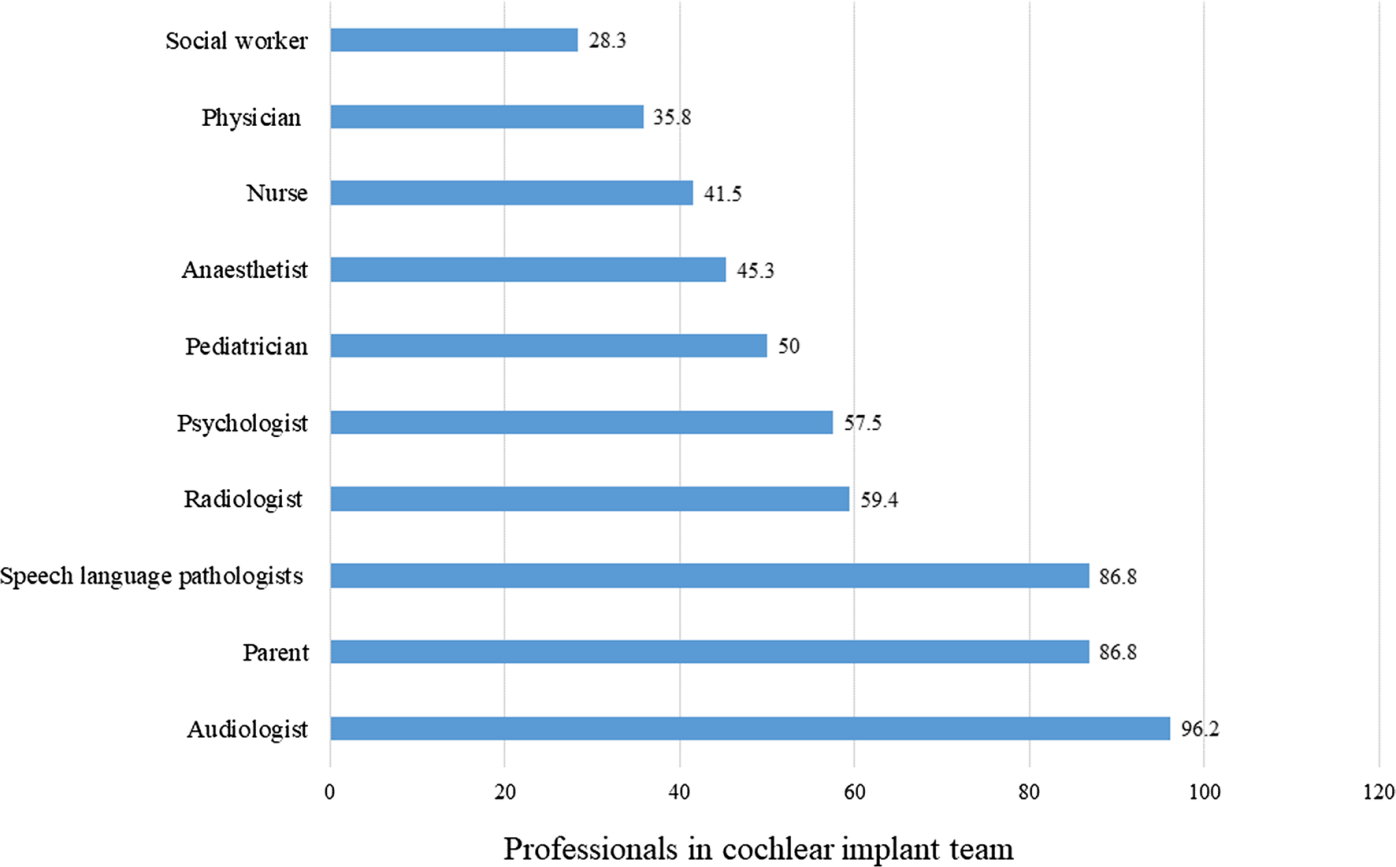
Professionals in cochlear implant team

Figure 2 illustrates the other professionals in a cochlear implant team. The most common professional was audiologist (96.2%) followed by parent (86.8%) and speech language pathologist (86.8%). Most otorhinolaryngologists mentioned about six (average − 5.88 ± 2.45) professionals as a part of the team.

The most common sources of additional information on cochlear implantation were national and international conferences (63.2%) and articles and books (63.2%) followed by professional education (61.3%) and other sources as shown in Table 2.

**Table 2:** Sources of additional information on cochlear implantation

Sources	n (%)
National and international conferences	67 (63.2)
Articles and books	67 (63.2)
Professional education	65 (61.3)
Training events	45 (42.5)
Internet	35 (33)
Conversations with colleagues	29 (27.4)
Education by industry partners/manufacturer-supported events, visits from manufacturer representatives	28 (26.4)

The final question was an open-ended question that asked the otorhinolaryngologists to indicate the challenges in cochlear implantation in India. Most of the respondents gave multiple challenges. Table 3 depicts the challenges in cochlear implantation in India as per the participants.

**Table 3:** Depicts the challenges in cochlear implantation in India as per the participants

Challenges in cochlear implantation	No. of participants
High costs, financial burden	52
Lack of awareness among parents/masses	20
Lack of mentorship to aspiring surgeons	16
Issues with availability of rehabilitation/specialized services	16
Late/delay in identification of hearing loss	12
Lack of trained manpower	8
Delays due to government policies/approvals	5
Lack of awareness among professionals	8
Stigma/social issues	5
Issues with accessibility	5
Less motivation/willingness among parents	5
Issues with maintenance of implant	5
Lack of support	2

## Discussion

The survey included questions based on knowledge regarding suitability of cochlear implantation and various schemes available from government. Further, beliefs and practices towards cochlear implants were explored followed by challenges in cochlear implantation in India. One hundred and six otorhinolaryngologists participated in the survey of which 62 were males and 44 females. A quarter of the otorhinolaryngologists performed cochlear implantation with a surgical experience of 2–500 surgeries.

Cochlear implantation surgery and rehabilitation is supported under the Assistance to Disabled Persons for Purchase/Fitting of Aids and Appliances (ADIP) scheme since the year 2014. Under this scheme, 0.6 million INR per unit is borne by the Government of India towards cochlear implantation for children from families of low socioeconomic background [12]. Rashtriya Bal Swasthya Karyakram (RBSK) supports CI for only children bellow 2 years of age. The other schemes mentioned in the options namely National Programme for Prevention and Control of Deafness, and Sound hearing initiative do not have a provision for supporting surgery for cochlear implantation. In the present study, only 55.7% ENTs correctly identified the Assistance to Disabled Persons for Purchase/Fitting of Aids and Appliances (ADIP) scheme as the program by the Government of India supports cochlear implantation.

Studies over several decades have highlighted the importance of reducing the age of implantation among the children born with hearing impairment to improve their speech and language learning outcomes [13–16]. In the next question, the respondents were asked to identify the lowest age for paediatric cochlear implant for children with bilateral, profound sensorineural hearing loss according to U.S. Food and Drug Administration. In 2020, Cochlear Limited, received the U.S. Food and Drug Administration approval for lowering the age to 9 months. This was made with an aim to improve the hearing abilities of children born with hearing impairment and provide them with a speech and language learning trajectory like their hearing peers [2]. Scattered responses were obtained for the lowest age for paediatric cochlear implant and as the guidelines for the lowest age for cochlear implantation are evolving professionals involved need to keep updating themselves.

In terms of the beliefs towards cochlear implants, most otorhinolaryngologists agreed that Cis need regular maintenance and adjustments. These findings are similar to a previous study in a multi-country study among otorhinolaryngologists [17]. Further, about 47% of them did not consider cochlear implant as a hindrance during sport activities, 25.5% were neutral and 25.5% considered them as a hindrance. D’Haese et al. [17] reported that 84% German otorhinolaryngologists agreed while 60–70% respondents from other nations agreed to the same. Most of the otorhinolaryngologists (86.8%) disagreed that qualitatively there is difference between hearing aids and cochlear implants. Only a few otorhinolaryngologists agreed that there is no difference between the two amplification devices. Very few otorhinolaryngologists in the multi-national study [17] agreed that there is no difference between the two amplification devices. In the final item of this sub-section, otorhinolaryngologists were asked to indicate if they felt that additional training is required in dealing with cochlear implant recipients. 82% ENTs felt the need of having additional training for dealing with cochlear implants.

As per the Centre for Disease Prevention and Control guidelines, children younger than two years of age with cochlear implants should receive pneumococcal vaccines as per the immunization schedule. Further, all children and adults undergoing cochlear implant surgery should complete their pneumococcal vaccine schedule, two weeks before the surgery [18, 19]. Recent consensus studies to establish clinical guidelines in Indian context have also emphasized on the need to complete pneumococcal vaccine schedule prior to cochlear implantation in children and adults [20, 21]. In the present study, more than 60% of the otorhinolaryngologists correctly indicated the recommended duration of 2 weeks before surgery.

The use of antibiotic prophylaxis both pre-operatively and post-operatively has also been recommended [22]. A study in otorhinolaryngologists in United Kingdom involved in implant surgery revealed that all of them followed an antibiotic protocol. Intravenous antibiotics were given either once (55%) or thrice (45%) at perioperatively (85%) or at induction (15%). Further, 45% otorhinolaryngologists prescribed oral antibiotics while 25% otorhinolaryngologists prescribed single dose of intravenous antibiotics without any subsequent oral antibiotics [23]. In the present study, 63.2% recommended use of one single intravenous antibiotic dose.

The candidacy criteria for cochlear implantation keeps evolving with advancements in technology. However, overall, the candidacy criteria involve exploration of medical status, benefit for communication and required support on the psychological, educational, family, and rehabilitative situation [1]. The otorhinolaryngologists were asked to indicate the factors considered while deciding the ideal candidate for cochlear implantation. The most common factor indicated was degree of hearing loss (80.2%) followed by limited benefit from hearing aids (73.6%).

A test battery approach is followed using several preoperative assessments which include both objective and subjective tests. The commonly included test by the otorhinolaryngologists was pure tone audiometry followed by auditory brainstem response and computerized tomography scan. In a survey among audiologists in the US working with cochlear implants in adults, most of the respondents used speech-based test materials during pre-operative testing. These included minimum speech test battery, AzBio sentences in quiet, consonant vowel nucleus consonant monosyllabic test [24]. Interestingly, in the present study, more than 60% otorhinolaryngologists included speech-language assessments and psychological evaluation. McRackan et al. [25] have emphasized on the importance to measure and include patient expectations during pre-operative counselling. Again, as the candidacy criteria for cochlear implantation keeps evolving, there is a need to adapt and improvise the include additional assessment procedures along with the traditional ones.

In the next question, the otorhinolaryngologists were asked to indicate the three most important steps in cochlear implantation. Based on the responses, rehabilitation, surgery, and ECAP responses emerged to be of most importance. These findings are in consensus with a previous multi-national survey among otorhinolaryngologists from where ‘rehabilitation’ was rated first [17, 26]. In both the previous surveys, the second most important was hearing preservation as compared to surgery in the present study.

Most of the otorhinolaryngologists felt that they play an important role during switch-on. Further, varied responses were obtained for duration of follow-up sessions. Vaerenberg et al. [27] in their global survey involving 17 countries reported usual trend for follow-up sessions was 3 in first quarter, 3 in following 3 quarters and 1 annual session. Again, variability was seen in the wait period before the switch on. This variability is also seen in the clinical practice guidelines for cochlear implants [28]. Cochlear implantation and further rehabilitation involve key-roles played by several team members in a multi-disciplinary team. As rightly pointed out by the otorhinolaryngologists in the present survey, audiologists, parents, and speech language pathologists are the key members.

Otorhinolaryngologists reported attending national and international conferences, reading articles and books and professional education to be the top sources of additional information on cochlear implantation. The least likely sources were education by industry partners/manufacturer-supported events, visits from manufacturer representatives. These findings are similar to previous survey in otorhinolaryngologists [17]. The final open-ended question elicited responses from the otorhinolaryngologists on the challenges in cochlear implantation in India. The responses were analysed thermically to identify the board themes. The most common responses indicate towards high costs and financial burden. Studies among healthcare professionals have indicated barriers such as limited knowledge of cochlear implants and eligibility criteria, referral process [29, 30]. The findings of the survey indicate an overall positive belief and practices towards cochlear implantation by otorhinolaryngologists in India. However, there is a need to spread more knowledge and awareness among them about the recent advances and schemes that would further improve their service delivery. The additional information can be provided in the form additional training related to CI, national and international conferences, recent articles and books.
